# Optimism and Hope in Chronic Disease: A Systematic Review

**DOI:** 10.3389/fpsyg.2016.02022

**Published:** 2017-01-04

**Authors:** Cecilia C. Schiavon, Eduarda Marchetti, Léia G. Gurgel, Fernanda M. Busnello, Caroline T. Reppold

**Affiliations:** Psychological Assessment Laboratory, Department of Health Sciences, Federal University of Health Sciences of Porto Alegre (UFCSPA)Porto Alegre, Brazil

**Keywords:** optimism, hope, chronic disease, randomized controlled trial, therapy

## Abstract

There is a growing recognition that positive psychological functioning (which includes constructs such as optimism and hope) influences health. However, the understanding of these underlying mechanisms in relation to health is limited. Therefore, this review sought to identify what the scientific literature says about the influence of optimism and hope on chronic disease treatment. A search was conducted in the PsycINFO, Scopus, Pubmed, and Web of Science databases using the indexing terms *optimism, hope, chronic diseases, randomized controlled trial*, and *treatment* between 1998 and 2015. In the articles, we identified the most studied diseases in context, the assessment instruments used, the participant characteristics investigated, the results found, and the publication dates. From our analysis of the articles that met our inclusion criteria, it appears that the study of these constructs is recent and there is evidence that individuals with greater optimism and hope seek to engage in healthier behaviors, regardless of their clinical status, and that this contributes to chronic disease treatment. More research is needed so that targeted interventions can be carried out effectively in chronic disease treatment.

## Introduction

The study of hope and optimism has grown in recent decades with the emergence of Positive Psychology. In an article entitled “Positive Psychology: An Introduction,” Seligman and Csikszentmihalyi (2000) suggest that the field of psychology up to the end of the 1990s primarily considered the issues of healing and damage repair and pointed out that attributes such as hope, wisdom, creativity, courage, spirituality, responsibility, and perseverance were being ignored by professionals and researchers in the field. Citing this gap in knowledge, these authors suggested that researchers also consider the individual's strengths and virtues as the twenty-first century began. Thus, studies in Positive Psychology were initiated, evaluating the subjective experiences of individuals regarding the past (well-being contentment and satisfaction), the present (flow and happiness), and the future (hope and optimism; Seligman and Csikszentmihalyi, 2000).

Currently, the overriding theme that has emerged after more than a decade of research in Positive Psychology is what Seligman (2008) calls “positive health.” According to the author, mental health (which consists of positive emotions, engagement, purpose, relationships, and positive achievements) is something greater than the mere absence of mental illness. It is quantifiable and predictive and leads to greater achievement, lower depression levels, and better physical health. Such definition is in agreement with the World Health Organization (WHO), cited by Segre and Ferraz (1997), which defines health not only as the absence of disease, but as a condition of perfect physical, mental, and social well-being. It therefore increases longevity, reduces healthcare costs, and leads to improvements in mental health in relation to aging and disease prognosis (Seligman, 2008).

The impacts that the constructs optimism and hope can have on physical health have been discussed for some time and it is suggested that they can bring relevant results for health in general and for physical well-being (Snyder et al., 1991b). In this context, Snyder et al. (1991a) defined hope as a state of positive motivation based on three components: objectives (goals to be achieved), routes (planning to achieve these goals), and agency (motivation directed toward these objectives). More recently, Kortte et al. (2012) added that hope represents a patient's sense of determination to achieve his/her objectives (Snyder et al., 1991a,b; Kortte et al., 2012).

In regards to optimism, Scheier and Carver (1985) defined it as an overall tendency to believe that vivid experiences will lead to good results rather than bad ones. Carver et al. (2010) explained that to be optimistic is to maintain a generally favorable expectation about the future. Hart et al. (2008) added that overall positive expectations are considered one of the main determinants for knowing whether people will continue to pursue their life objectives in a condition of chronic disease (Scheier and Carver, 1985; Hart et al., 2008; Carver et al., 2010).

There is evidence that optimism motivates the individual to take proactive measures to protect his/her health, while pessimism is associated with behaviors that are adverse to health (Carver et al., 2010). On the other hand, studies such as those by Cohen et al. (1999) and Segerstrom (2005) have shown that when stressors are short-lived (i.e., less than a week) optimism appears to be protective against the effects of stress. However, this effect is reversed when the stressors are prolonged, as optimists are more immunologically vulnerable under such circumstances (Cohen et al., 1999; Segerstrom, 2005; Carver et al., 2010).

Evidence from the meta-analysis performed by Rasmussen et al. (2009) suggests that optimism is a significant predictor of positive results for physical health. Furthermore, a review by DuBois et al. (2012) reports that there is significant evidence of positive psychological attributes (especially optimism) being associated with better cardiac outcomes. However, differently from these articles, the present review aims to make a qualitative analysis, investigating the constructs optimism, and hope specifically, not as positive psychological attributes in general, focusing on multiple outcomes (Rasmussen et al., 2009; DuBois et al., 2012).

Given the above information, we aimed to conduct a literature review on the influence of optimism and hope on the prevention and treatment of chronic disease, identifying the most studied diseases, the assessment instruments used, the investigated participants' characteristics, the results, and the evolution of the research in this context.

## Methods

### Search strategy

We searched the Scopus, Pubmed, Web of Science, and PsycINFO databases. The search was conducted using the following terms: “optimism,” “hope,” “chronic disease,” “randomized controlled trial,” and “therapeutics” (and their equivalents in Portuguese according to the DeCS structured and trilingual vocabulary—descriptors in the health sciences, except the term “optimism,” which is not indexed). The standard search strategy was hope* OR optimis* AND “randomized controlled trial” AND “chronic disease” AND therap* and NOT review, including articles from 1998 (the year Martin Seligman began the Positive Psychology movement) to March 2015.

### Study eligibility

The present review included all studies whose participants had a chronic disease, regardless of age, and which were preferably randomized controlled trials and had optimism, hope, or both constructs as part of the study. Excluded were book chapters; studies not published in Portuguese, English, or Spanish; review studies and studies with animal models; summaries of scientific events; dissertations and theses; and unpublished studies.

According to Escosteguy (1999), studies with a randomized design are the best source of scientific evidence available and the best source for determining an intervention's efficacy. As such, they are considered the gold standard of scientific research. Nevertheless, even though the descriptor “randomized controlled trials” was used in the search strategy, we decided to include articles with other designs in order to include a greater number of publications, as this is a recent subject and publications in the field are scarce. To build the results, only studies with a randomized controlled design were evaluated for their quality (Escosteguy, 1999).

We initially analyzed the titles and abstracts of all the articles originating from the searches performed in the previously cited databases. All studies that met the inclusion criteria were selected for evaluation of their full text and for subsequent extraction of relevant data. For both, the article selection and the text analysis, two reviewers participated in collecting and assessing data from the complete articles, and made their selections according to the eligibility criteria. Standardized forms were used to extract relevant data from each study. These forms were prepared specifically for the present review.

### Analysis of study quality

For the studies with a randomized controlled design, quality assessment was performed according to the following main aspects: secrecy of the allocation list; analysis by intention to treat; baseline comparability; blinding of the outcome assessment, and description of losses and exclusions. The absence of a description such as how the allocation list was generated was considered the absence of allocation secrecy. The quality analysis was also based on the GRADE approach, as recommended by the Cochrane Collaboration (Guyatt et al., 2008) for the analysis of study quality. The data analysis was descriptive of the studies' results. The methodological characteristics and principal evidence were reported according to the studies' main objectives (Guyatt et al., 2008).

## Results

The analysis enabled us to evaluate which diseases were studied more in terms of the constructs of optimism and hope, the most used scales, the most studied groups (sex and age of the assessed group), the year of the studies, and in which cases the results proved to be most effective. In the initial search, 2464 articles were found that could be potentially included and, after reading their titles and abstracts, repeated articles, those that did not address the proposed topic and those that did not meet the inclusion criteria were excluded. We believe that the large number of articles found in the initial search was due to the fact that the term “optimism” is not indexed in the DeCS and because the word “hope” has a wide range of meanings.

**Figure 1:**
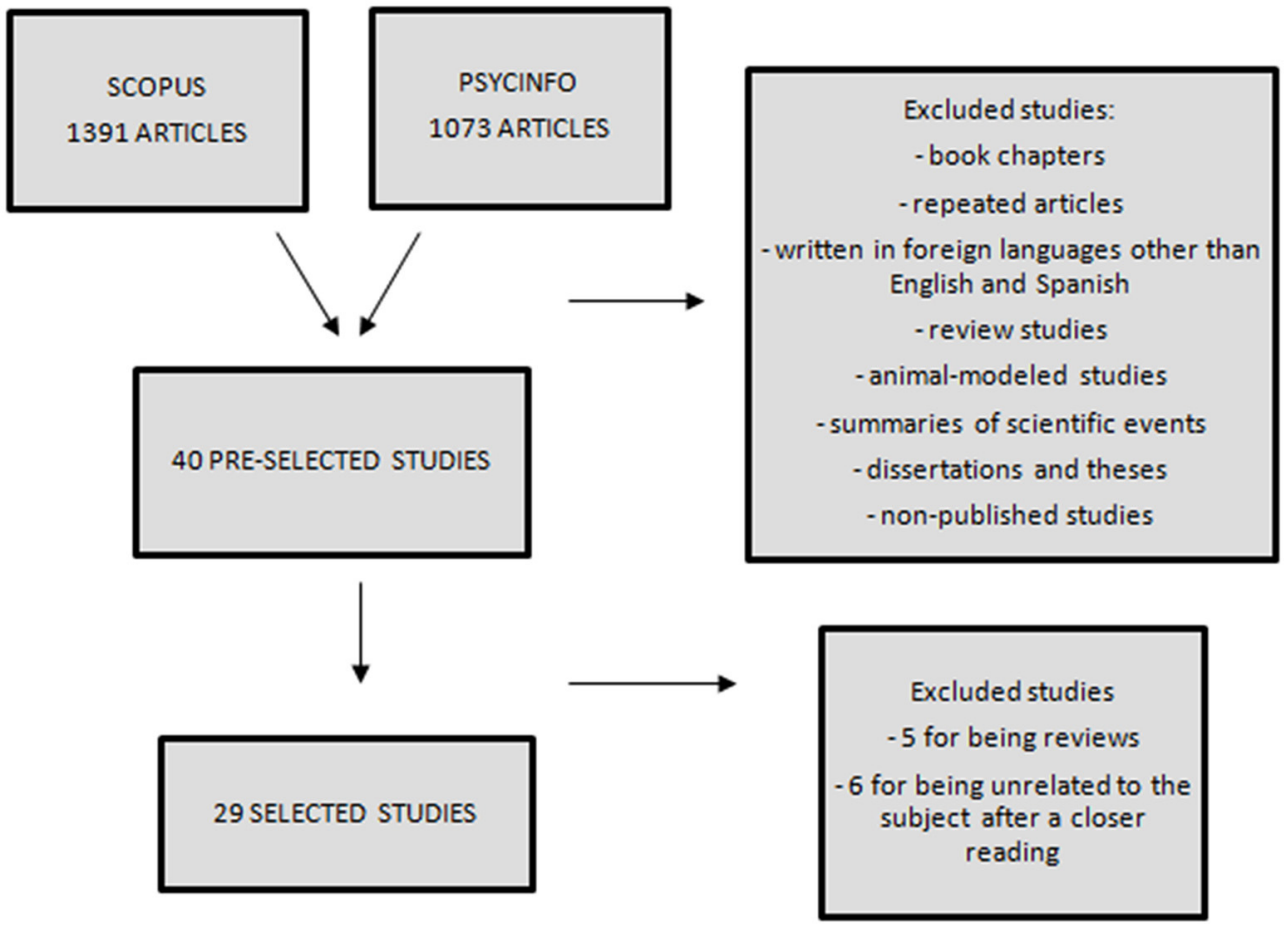
**Flow chart of included and excluded studies**.

Only 40 studies were ultimately selected and, of these, eleven were excluded as five of them were reviews and six were found not to be related to the topic upon a closer reading. Of the 29 articles found, eight were selected from the PsycINFO database, eight from Scopus, and 13 from additional references found in the pre-selected articles (Figure 1). Although, the Pubmed and Web of Science databases did not yield results that met the inclusion criteria, the PsycINFO and Scopus searches yielded a wide range of articles. We found 1073 articles in PsycINFO and 1391 articles in Scopus. In regard to their designs, nine of the studies were randomized controlled trials (Table 1) and 20 had various other designs (Table 2). The specific characteristics of each study are shown in Tables 1, 2.

**Table 1:** **Randomized controlled trials**.

**Author/Year**	**Disease**	**Design**	**Construct**	**Scale**	**Sample**	**Results**
Giltay et al., 2004	Cardiovascular disease	Randomized controlled trials	Optimism	Dutch scale of subjective and well-being for older persons, with a subscale for optimism	(*n* = 999, 65–85 years old) Random sample (stratified by age and sex) of people who live in Arnhem, Netherlands	There is a relationship of graduated and independent protection between dispositional optimism and all causes of mortality in old age
Matthews et al., 2004	Carotid atherosclerosis	Randomized controlled trials	Optimism	Life Orientation Test (LOT, Scheier and Carver, 1985)	(*n* = 209, 42–50 years old) Healthy, middle-aged pre-menopausal women	Optimistic women are less likely to show progression of carotid artery disease in middle age than pessimistic women
Hart et al., 2008	Multiple sclerosis	Randomized controlled trials	Optimism	The Life Orientation Test-Revised (LOT-R; Scheier et al., 1994)	(*n* = 127, 18 years old or over) Multiple sclerosis patients who participated in another randomized clinical trial and were evaluated at the beginning, middle, and end and at 6 and 12 months after the therapy	Optimism helps people to achieve their life objectives, even in the face of difficulties. Increased optimism is a mechanism that appears to assist multiple sclerosis patients in increasing their benefits over time
Geers et al., 2010	Pain tolerance, vascular response, and pulse excitability	Randomized controlled trials	Optimism	The Life Orientation Test-Revised (LOT-R; Scheier et al., 1994)	(*n* = 116, 18–45 years old) Adults without histories related to chronic pain	Optimism was associated with lower pain classifications in the placebo condition
Saquib et al., 2011	Breast cancer	Randomized controlled trials	Optimism	The Life Orientation Test-Revised (LOT-R; Scheier et al., 1994)	(*n* = 2967, 18–70 years old) Women in the initial stage of breast cancer	Optimism is not associated with breast cancer or death from all causes in the multivariate analysis
Boehm et al., 2013	Hypertension, stroke, diabetes, and heart disease	Randomized controlled trials	Optimism	The Life Orientation Test (LOT, Scheier and Carver, 1985)	(*n* = 990, mean age of 55.1 years old) American people, mostly white	Optimism is associated with healthier lipid profiles (higher HDL levels and lower triglyceride levels)
Richman et al., 2005	Hypertension, diabetes mellitus, and respiratory tract infections	Randomized controlled trials	Hope	Emotion Scale (Ellsworth and Smith, 1988)	(*n* = 5500, 55–69 years old) Patients randomly selected based on data from a multi-disciplinary practice	Higher levels of hope are associated with a decline in probability of having or developing a disease
Warber et al., 2011	Coronary syndrome	Randomized controlled trials	Hope	Hope Scale (Snyder et al., 1996)	(*n* = 41, 25–75 years old) Men and women of any ethnic/racial group with a history of unstable angina or heart attack for 6–18 months before the intervention	Hope is inversely correlated to stress and depression. An association was also observed between hope and positive cardiovascular outcomes
Gelkopf et al., 2013	Chronic combat-related post-traumatic stress	Randomized controlled trials	Hope	Hope Scale (Snyder et al., 1991a)	(*n* = 22, 24–59 years old) War veterans with chronic combat-related post-traumatic stress who participated in the 1-year Nature Adventure Rehabilitation intervention	This study's results suggest that the 1-year NAR intervention leads to significant improvements in daily functioning and hope

**Table 2:** **Other designs**.

**Author/Year**	**Disease**	**Design**	**Construct**	**Scale**	**Sample**	**Results**
Shepperd et al., 1996	Heart disease	Longitudinal study	Optimism	Life Orientation Test (LOT; Scheier and Carver, 1985)	(*n* = 22, 40–80 years old) Patients who participated in an 18-week cardiac rehabilitation program	Optimism measured at the beginning of the program was associated with greater success in achieving lower saturated fat, body fat, and overall coronary risk levels as well as greater success in increasing aerobic capacity by the end of the program
Scheier et al., 1999	Coronary artery bypass surgery	Prospective cohort study	Optimism	Life Orientation Test-Revised (LOT-R; Scheier et al., 1994)	(*n* = 309, mean age: 63 years old) Patients scheduled to undergo elective coronary artery bypass surgery in a hospital in Pittsburgh, PA	Optimistic people were significantly less likely to be hospitalized again for a wide range of aggregated problems
Kubzansky et al., 2001	Heart disease	Longitudinal cohort study	Optimism	Optimism–Pessimism Scale	(*n* = 162, 21–80 years old) Cases of incident coronary heart disease: 71 cases of non-fatal myocardial incidents, 31 cases of fatal coronary heart disease and 60 cases of angina pectoris	An optimistic outlook can protect against coronary heart disease risk in older men
Kubzansky et al., 2002	Chronic airway obstruction	Prospective cohort study	Optimism	Revised Optimism-Pessimism Scale (PSM–R-Malinchoc et al., 1995)	(*n* = 2280, 21–80 years old) Men residing in the Greater Boston region	Optimism is linked to higher pulmonary function levels and lower rates of pulmonary function decline in older men—a protective effect, regardless of smoking habits
Allison et al., 2003	Head and neck cancer	Prospective cohort study	Optimism	French version of the Life Orientation Test	(*n* = 101, mean age: 58.3 years old) Patients with cancer diagnosed between March 1, 1997 and August 31, 1998 at the Centre Hospitalier Universitaire, Clermont-Ferrand, France	Optimism provides 1 year of survival independent of other socio-demographic and clinical variables
Urcuyo et al., 2005	Breast cancer	Cross-sectional study	Optimism	Life Orientation Test-Revised (LOT-R; (Scheier et al., 1994))	(*n* = 230, 27–87 years old) Patients in the initial breast cancer stage recruited through various medical practices in the Miami area and at the local American Cancer Society (ACS) office	Benefits to the condition were related to a disposition to be optimistic about life
Pinquart et al., 2007	Cancer	Cross-sectional study	Optimism	The Life Orientation Test (LOT, Scheier and Carver, 1985)	(*n* = 153, mean age: 57.7 years old) Participants recently diagnosed in two oncology wards of a German oncology and hematology clinic	Optimistic cancer patients may interpret their illness less negatively, put negative feelings aside, better manage stressors, focus on ways to deal with problems and may therefore suffer fewer negative changes
Bargiel-Matusiewicz and Krzyszkowska, 2009	Rheumatoid arthritis, back pain, or neuropathy	Cross-sectional study	Optimism	The Life Orientation Test-Revised (LOT-R; Scheier et al., 1994)	(*n* = 62, 24–65 years old) Patients at rehabilitation centers in the cities of Warsaw and Radomsk suffering from rheumatoid arthritis, back pain, or neuropathy	A highly optimistic life orientation was related positively with pain control variables and negatively with catastrophic attitudes
Flett et al., 2011	Crohn's Disease and ulcerative colitis	Cross-sectional study	Optimism	Life Orientation Test (Scheier and Carver, 1985)	(*n* = 51, mean age: 37.7 years old) Participants were recruited at St. Michael's Hospital in Toronto, Ontario, of which 27 had Crohn's Disease and 24 had ulcerative colitis	Higher levels of optimism were associated with lower levels of perfectionism. Thus, optimistic people generally did not use emotional worry or maladaptive attitudes in relation to chronic diseases
Johnson et al., 2001	HIV	Prospective longitudinal study	Hope	Beck Hope Scale (BHS; Beck et al., 1974)	(*n* = 103, mean age: 38 years old) HIV-positive men + homosexuals	In HIV-positive people, an increase in hopelessness predicted an increase in depression after controlling for social support
Hartley et al., 2008	Knee and hip replacement surgery	Longitudinal study	Hope	Hope Scale (Snyder et al., 1991a)	(*n* = 100, mean age: 64 years old) Older adults residing in an orthopedic clinical community	Hope is a significant predictor of pre-surgical depression but is not predictive of depression or functional ability after surgery
Kortte et al., 2010	Spinal cord injury	Prospective study	Hope	Hope Scale (Snyder et al., 1991a)	(*n* = 87, 18–85 years old) Adults who participated in an acute spinal cord rehabilitation program in two metropolitan hospitals	There was a link between hope and greater life satisfaction during the initial period of acute rehabilitation
Kortte et al., 2012	Spinal cord dysfunction, stroke, amputation or orthopedic surgery recovery	Longitudinal study	Hope	Hope Scale (Snyder et al., 1991a)	(*n* = 174, mean age: 57 years old) Adults who participated in a rehabilitation program for spinal cord dysfunction, stroke, amputation or orthopedic surgery recovery	Interventions aimed at increasing the hope of individuals can be useful for increasing participation in improved outcomes following an acute medical rehabilitation
Halding and Heggdal, 2012	Chronic obstructive pulmonary disease	Qualitative study	Hope	Thirty-six individual qualitative interviews	(*n* = 18, 52–81 years old) Patients recruited at pulmonary rehabilitation centers in Norway	During rehabilitation, knowledge of opportunities for health and well-being, engagement in self-management and hope for the future were strengthened, as well as healthy life transitions
Waynor et al., 2012	Mental illness	Cross-sectional study	Hope	The State Hope Scale (SHS; Snyder et al., 1996)	(*n* = 74, 23–63 years old) Individuals recruited from five “employment support” programs in a Northeastern Brazilian state	Hope and symptoms are inversely related
Hawro et al., 2014	Psoriasis	Cross-sectional study	Hope	Basic Hope Inventory (Trzebiński and Zięba, 2003)	(*n* = 60, mean age: 46.8 years old) Patients with psoriasis hospitalized in the Department of Dermatology, Pediatric Dematology and Dermatologic Oncology at the Lodz Medical University	Higher levels of hope are correlated with better life quality
Scioli et al., 1997	Chronic disease, general health	Prospective cohort study	Optimism and hope	Life Orientation Test (LOT, Scheier and Carver, 1985) and (the Hope Scale; Gottschalk, 1974, 1985)	(*n* = 57, mean age: 19.5 years old) Students recruited from psychology courses at a small New England college	Less optimism is correlated to reports of more severe disease 10 weeks later. Less hope is correlated with a higher incidence of disease and reports of greater severity of total diseases
Hou et al., 2010	Cancer	Prospective cohort study	Optimism and hope	Chinese version of the Life Orientation Test and the Hope Scale	(*n* = 234, 29–82 years old) Chinese patients with colorectal cancer	Maintenance or adoption of optimistic personalities is associated with recovery from emotional anguish and preservation of resilience
Schöllgen et al., 2011	General chronic disease	Cross-sectional study	Optimism and hope	Hope Scale (Snyder et al., 1991a), Optimism–Affective valence of future perspectives scale (Brandtstädter and Wentura, 1994)	(*n* = 2454, 40–85 years old). The data were extracted from the German Aging Survey	Psychological resources positively affected health in all groups
Lopez-Vargas et al., 2014	Chronic kidney disease	Focus group	Optimism and hope	Focus group	(*n* = 38, 18 years old or older) People with chronic kidney disease (stages 1–4). The sample is from three hospitals in Sydney, Australia	Optimism and a positive perspective on life and its circumstances enabled the patients to deal with their disease and take full advantage of their lives

One can observe that most publications addressing the relationship between hope/optimism and chronic disease were published from 2005 onwards. In addition, the most researched diseases were: heart disease (six studies), cancer (five studies), respiratory tract diseases (three studies), spinal cord diseases (two studies), hypertension (two studies), stroke (two studies), and diabetes (two studies). Other researched diseases included multiple sclerosis, psoriasis, Crohn's disease, ulcerative colitis, and others (Tables 1, 2), which corroborated data from the PNAD (2002), cited by Almeida et al. (2002) on the most prevalent diseases in the population (Almeida et al., 2002).

The most used instruments in these studies to assess optimism were the Life Orientation Test (LOT; Scheier and Carver, 1985)—which was used to assess the construct in six of the 15 articles—and the Life Orientation Test–Revised (LOT-R; Scheier et al., 1994) which was also used in six of the 15 studies. Other scales used for this construct were: the Revised Optimism-Pessimism Scale (Kubzansky et al., 2001, 2002) and the Dutch Scale of Subjective Well-being for Older Persons (Giltay et al., 2004). The Hope Scale (Snyder et al., 1996) was the most used instrument to evaluate hope in the studies (six out of ten). Other evaluation methods were: the Emotion Scale (Richman et al., 2005), individual qualitative interviews (Halding and Heggdal, 2012), the Beck Hopelessness Scale (Johnson et al., 2001), and the Basic Hope Inventory (Hawro et al., 2014). As for the studies that addressed both constructs, the following instruments were used: the Hope Scale Optimism–Affective valence of future perspectives scale (Schöllgen et al., 2011); the Life Orientation Test, the Gottschalk Hope Scale (Scioli et al., 1997); and the Chinese version of the Life Orientation Test and Hope Scale (Scheier and Carver, 1985; Scheier et al., 1994; Snyder et al., 1996; Scioli et al., 1997; Johnson et al., 2001; Kubzansky et al., 2001, 2002; Giltay et al., 2004; Richman et al., 2005; Hou et al., 2010; Schöllgen et al., 2011; Halding and Heggdal, 2012; Hawro et al., 2014).

All of the research subjects evaluated in the articles were adults between the ages of 18 and 85. Fourteen of the 29 studies evaluated people who were receiving treatment for their conditions at a clinic or hospital while seven out of 19 articles about optimism and 3 from 14 articles about hope evaluated healthy individuals. Some association between higher hope/optimism levels and a healthier profile was observed in 27 of the 29 studies. In regard to the results perceived by the study participants after intervention, only two articles found no relationship between the constructs and relevant results.

In an extended analysis of the studies, it is clear that the people with heart disease had the most significant results for their physical health. As for the other diseases, the results were more subjective, as the outcome in many of the articles was suffering from fewer negative changes. In terms of analyzing the quality of the randomized controlled trials (Table 3), the main difficulty in most of the studies was related to allocation secrecy and blinding, but even though the studies conducted by randomized controlled trial design were under more rigorous standards, their results did not differ significantly from other studies' designs.

**Table 3:** **Methodological quality of the studies included with randomized clinical design**.

	**Randomization**	**Allocation secrecy**	**Analysis by treatment intention**	**Baseline Comparability**	**Blinding**	**Description of losses and inclusions**
Giltay et al., 2004	A	I	I	A	I	A
Matthews et al., 2004	A	I	I	A	A	I
Hart et al., 2008	A	A	A	A	A	A
Geers et al., 2010	A	A	I	A	I	I
Saquib et al., 2011	A	I	I	A	I	I
Boehm et al., 2013	A	I	I	I	I	A
Richman et al., 2005	A	I	I	I	I	A
Warber et al., 2011	A	A	A	A	I	I
Gelkopf et al., 2013	A	I	A	A	I	A

*A - Adequate; I- Inadequate*.

## Discussion

The present study's results show that there is still a small number of articles that relate optimism and hope with chronic disease, that many of the authors have focused on studying mainly heart disease and cancer and that in comparison with hope, optimism is used is a larger number of studies. However, the quantity of studies about both constructs has grown due to an increasing interest by researchers in studying human potential. Hart and Sasso (2011) mapped the contours of contemporary Positive Psychology and reported significant growth in the number of publications addressing this subject in the last decade. They confirmed that researchers in this area have focused most of their attention on two of the three “pillars” of Positive Psychology, number (1) and (2), as proposed by Seligman and Csikszentmihalyi (2000): (1) the study of positive subjective experience and (2) positive personal characteristics (3) the study of positive institutions (Seligman and Csikszentmihalyi, 2000; Hart et al., 2008).

The main instruments used to assess positive personal characteristics were the Life Orientation Test (LOT; Scheier and Carver, 1985)—which was used in eight of the 19 articles that evaluated the construct—and the Life Orientation Test–Revised (LOT-R; Scheier et al., 1994), which was used in six of the 19 articles. The LOT (Scheier and Carver, 1985) is a self-reporting questionnaire composed of 12 items that aim to measure the life orientation construct in terms of how people perceive their lives in a more optimistic or less optimistic way. The instrument was revised in 1994 and two items were removed that did not explicitly focus on future expectations, thus creating the Revised Life Orientation Test (LOT-R) (Scheier and Carver, 1985; Scheier et al., 1994).

The studies about optimism had more similar outcomes than the hope studies due to the fact that they focused mainly on cancer and cardiac diseases, while hope studies mentioned a wider range of conditions. Studying the importance of positive psychological attributes on different diseases gives us a broad analysis of their effect in different situations but also does not have enough studies about each condition to bring us a reliable theoretical foundation that the construct is actually important to treatment.

In regards to optimism, most of the studies assessed the relationship between optimism and heart disease. The results showed that optimism was associated with greater success in heart conditions (Shepperd et al., 1996), less chances of being re-hospitalized for a wide range of aggregated problems (including myocardial infarction and coronary artery bypass surgery; Scheier et al., 1999), reduced risk of coronary disease in elderly people (Kubzansky et al., 2001), lower cardiovascular mortality in a sample of elderly people (Giltay et al., 2004), and less chances for women to experience carotid disease progression (Matthews et al., 2004; Shepperd et al., 1996; Scheier et al., 1999; Kubzansky et al., 2001; Giltay et al., 2004; Matthews et al., 2004).

Regarding cancer, it was found that optimism predicted a year of survival regardless of other socio-demographic and clinical variables in patients with head and neck cancer (Allison et al., 2003) and more abilities to manage stressors while less optimistic cancer patients experienced more negative psychological changes (Pinquart et al., 2007). Also, Allison et al. (2003) found that individuals who had pessimistic attitudes had poorer physical health, were more likely to suffer from depression and had higher mortality rates. Evidence has suggested that individuals who possess an inner will to achieve personal goals and expect the best possible results are able to live longer, healthier lives—a conclusion also reached by Urcuyo et al. (2005) (Allison et al., 2003; Urcuyo et al., 2005; Pinquart et al., 2007).

As for other clinical conditions, optimism was found to lead to positive physical health outcomes in multiple sclerosis patients because they begin to seek opportunities to change their disease experiences and prosper in the midst of adverse conditions (Hart et al., 2008), lower perfectionism and emotional worry levels and lower non-adaptative attitudes toward chronic diseases in patients with Crohn's disease and ulcerative colitis (Flett et al., 2011). Also, it contributes to lower rates of decline in pulmonary function (Kubzansky et al., 2002) and lower pain classifications because people with a positive attitude toward life have better physiological results for dealing with pain than do pessimists (Bargiel-Matusiewicz and Krzyszkowska, 2009; Geers et al., 2010). In addition, Boehm et al. (2013) also reported that optimistic people are better prepared to face the challenges of adopting healthy behaviors and maintaining a normal Body Mass Index (BMI) (Kubzansky et al., 2002; Hart et al., 2008; Bargiel-Matusiewicz and Krzyszkowska, 2009; Geers et al., 2010; Flett et al., 2011; Boehm et al., 2013).

In regard to hope, the most used instrument (in eight of the 13 studies) was the Hope Scale (Snyder et al., 1996). This scale asks participants to assess each of 12 items on a scale of 1 (definitely false) to 8 (definitely true), with total scores varying from 8 to 64, where higher scores indicate higher levels of hope (Snyder et al., 1996).

There was no mutual condition in the articles about hope, so this construct was found to lower the likeliness of being diagnosed with respiratory tract infections (Richman et al., 2005) and protect individuals against pain and depression due to the fact that hopeful individuals believe that their current circumstances are temporary and can be transformed into better conditions (Hartley et al., 2008). Furthermore, through an analysis of a lung rehabilitation program, Halding and Heggdal (2012) found that well-being and hope for the future were strengthened during this process, which motivated people to transition to healthier life habits (Richman et al., 2005; Hartley et al., 2008; Halding and Heggdal, 2012).

Other studies showed that hope contributed to increase life satisfaction in individuals with spinal cord injuries at the time they were admitted to acute rehabilitation services (Kortte et al., 2010) and lead to a consistency of positive effects on diseases that involve different physiological systems (e.g., cardiovascular, metabolic and respiratory; Richman et al., 2005). In addition, they suggest that these positive emotions may play a role in protecting against disease development (Richman et al., 2005; Kortte et al., 2010).

Other benefit findings were reported in the studies of Johnson et al. (2001), showing that social support for HIV-positive people is inversely related to depressive symptoms and a lack of hope; Waynor et al. (2012), demonstrating that hope and symptoms are inversely related; Hawro et al. (2014) reporting that higher hope levels are correlated with better life quality; and Warber et al. (2011), concluding through a spiritual intervention that hope is inversely correlated with stress and depression and associated with positive cardiovascular outcomes. Using another methodology, Gelkopf et al. (2013) conducted a study in which the rehabilitation team of the Israeli Defense Forces (IDF) ran a rehabilitation program through adventures in nature. The results suggest that there were great improvements in the daily functioning and in the hope levels of the veterans who had chronic combat-related post-traumatic stress disorder (Johnson et al., 2001; Warber et al., 2011; Waynor et al., 2012; Gelkopf et al., 2013; Hawro et al., 2014).

In terms of the simultaneous study of optimism and hope, a study by Hou et al. (2010) indicates that the maintenance or improvement of optimism and hope is associated with recovery from mental anguish and the preservation of resilience. Schöllgen et al. (2011) showed that psychological resources such as self-esteem, hope, and optimism positively affect health. Lopez-Vargas et al. (2014) studied a focus group and reported that the patients diagnosed with chronic kidney disease believed that a positive and optimistic perspective on life enabled them to better deal with their diseases and that hope for a better future led them to feel more encouraged to implement changes in their lifestyles (Hou et al., 2010; Schöllgen et al., 2011; Lopez-Vargas et al., 2014).

Regarding the results perceived by participants after interventions, only two articles did not find a relationship between the constructs and relevant results. In a study by Saquib et al. (2011), the multivariate analysis of social support, depression, insomnia, optimism, and hostility did not find a statistically significant association between optimism and additional breast cancer occurrences nor mortality from all causes. In addition, Hartley et al. (2008) did not observe any association between hope and functional abilities as hope only had beneficial effects at the beginning of the prothesis placement study. This is due to the fact that the participants initially experienced discomfort as they awaited their surgeries and this discomfort subsequently diminished, rendering the effects of hope less perceptible (Hartley et al., 2008; Saquib et al., 2011).

## Conclusion

The results of the studies presented in this analysis suggest that there is a close relationship between the constructs of optimism and hope and a reduction in the effects of chronic disease. However, it is important to highlight that the association between optimism or hope and physical health differs depending on the context of the disease and the subjects. Besides, there is a need for further studies on this subject, mainly about hope, due to the lack of studies on the variety of diseases related to this construct. Therefore, more studies are needed in order to describe the benefits that these attributes can bring to the health of individuals. Consequently, intervention studies have to be developed to guarantee the effective implementation of the findings.

Through a general analysis of these studies, one can observe that cardiac patients with higher levels of optimism attained better results in terms of their physical health. Results were more difficult to observe in cancer patients. However, it was reported that optimistic people suffered fewer negative changes in their condition. As for hope, the results were more subjective. People with higher levels of hope reported having higher life quality and satisfaction, but the only relevant physical results were positive physical outcomes reported by patients with cardiovascular disease.

For further research, it's suggested the development of more studies related to different diseases and more interventions in the context of optimism and hope. Additionally, it's recommended the development of children and adolescents interventions because there is a lack of research about these subjects in this field of study.

## Author contributions

CS and EM contributions to the conception and design of the work, constant revision, approval of published version, agreement to be accountable for all aspects of the work. LG, FB, and CR analysis, or interpretation of data for the work, constant revision, approval of published version, agreement to be accountable for all aspects of the work.

### Conflict of interest statement

The authors declare that the research was conducted in the absence of any commercial or financial relationships that could be construed as a potential conflict of interest.
